# Atherosclerosis Begins in Childhood: A Life-Course Perspective on Arterial Remodeling from the Bogalusa Heart Study

**DOI:** 10.1007/s11883-026-01476-2

**Published:** 2026-10-29

**Authors:** Ileana De Anda-Duran, Grecia García Flores, Lydia A. Bazzano, Elaine M. Urbina

**Affiliations:** 1 Epidemiology Department, Celia Scott Weatherhead School of Public Health and Tropical Medicine, Tulane University, 1440 Canal St., Suite 2044, New Orleans, 70112 LA USA; 2 Internal Medicine and Public Health Departments, and Peter O’Donnell Jr. Brain Institute, University of Texas Southwestern Medical School, Dallas, TX USA; 3 Preventive Cardiology, Cincinnati Children’s Hospital Medical Center, University of Cincinnati, Ohio Cincinnati, USA

**Keywords:** Carotid intima–media thickness, Bogalusa heart study, Subclinical atherosclerosis, Cardiovascular risk factors, Life-course epidemiology, Childhood obesity

## Abstract

**Purpose of Review:**

This review summarizes carotid intima–media thickness (cIMT) findings from the Bogalusa Heart Study (BHS) and examines how cardiovascular risk factors across the life course contribute to early subclinical atherosclerosis.

**Recent Findings:**

BHS studies consistently demonstrate that obesity, dyslipidemia, and SBP are independently associated with increased cIMT in young adulthood. Life-course analyses show that cumulative BMI and LDL-C exposure independently predict adult cIMT, with race-specific sensitive periods identified for obesity. Metabolically abnormal obesity confers greater vascular risk than metabolically healthy obesity. LDL-C and the total cholesterol/HDL-C ratio are the principal lipid determinants of carotid wall thickening, with associations strengthening from childhood through midlife. SBP dominates in cross-sectional models, while BMI shows more consistent independent effects beginning in childhood. Cardiometabolic clustering further amplifies vascular risk, and adolescence represents a key developmental window, with pediatric metabolic syndrome performing no better than BMI alone in predicting adult cIMT.

**Summary:**

BHS evidence supports the view that atherosclerosis is a progressive disease that begins in childhood and develops through the cumulative burden of risk factors over the life-course. Obesity, dyslipidemia, and elevated blood pressure each independently contribute to cIMT, and their clustering within individuals amplifies this effect, such that the combined sustained burden across the life-course is the strongest determinant of subclinical arterial remodeling. This underscores the importance of early, sustained prevention strategies that target modifiable CV risk factors beginning in childhood.

**Graphic Abstract:**

**Figure:**
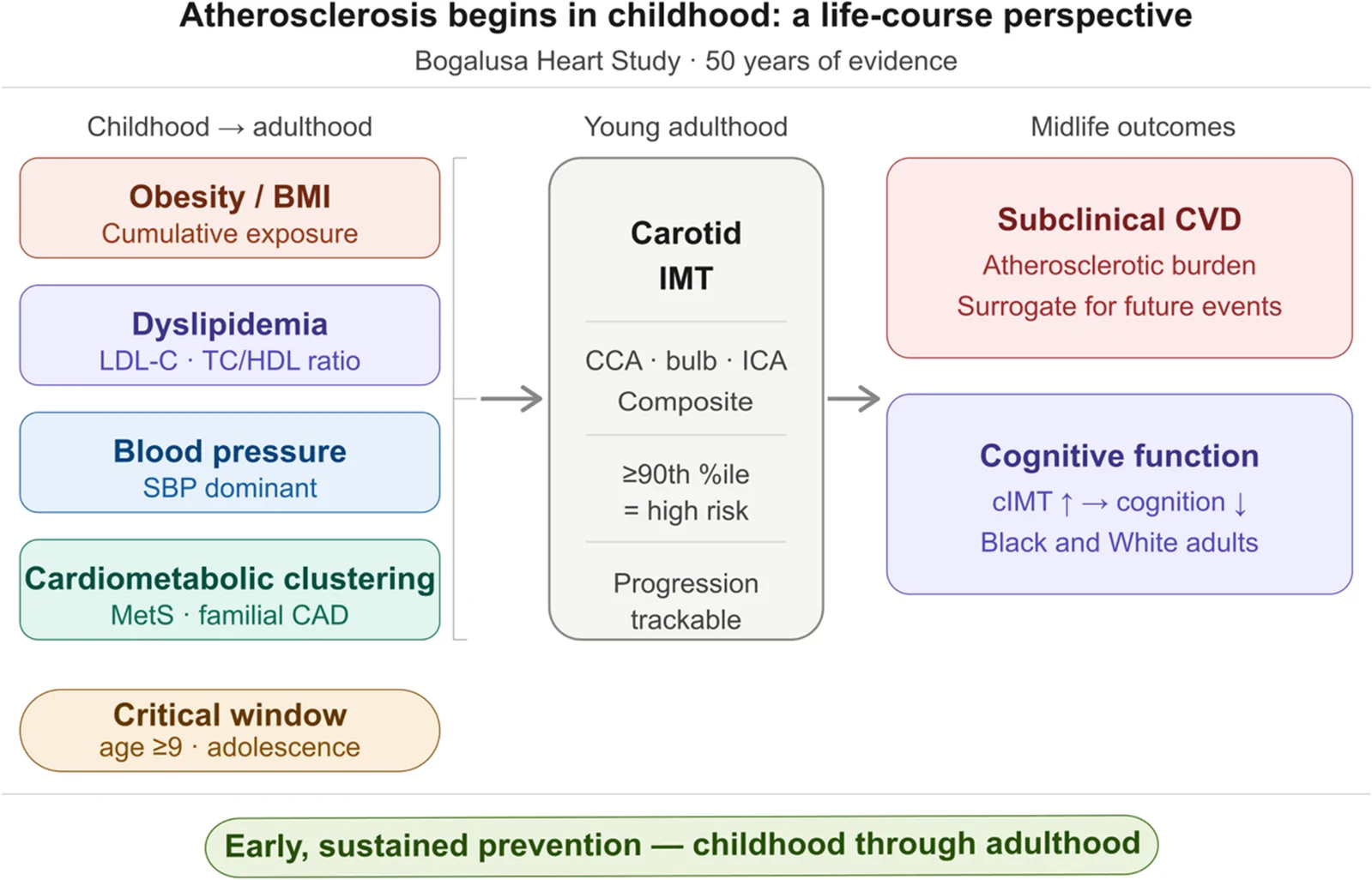
Cardiometabolic clustering: denotes the accumulation of multiple adverse cardiometabolic risk factors within an individual. Rather than representing a single risk factor, it serves as an integrative marker of cumulative cardiovascular risk, with vascular risk rising as adverse factors co-occur and compound.

## Introduction

Since its inception in 1973, the Bogalusa Heart Study (BHS) has been one of the few longitudinal cohort studies of Black and White individuals from rural Southern Louisiana, with the objective of identifying early-life risks and establishing the life-course nature of cardiovascular (CV) disease. The BHS has been instrumental in understanding how subclinical changes, such as subclinical atherosclerosis, are linked to its exposure beginning in childhood [1–3]. Findings have demonstrated that childhood risk factors are associated with both early vascular dysfunction and structural arterial changes, and that atherosclerosis begins early in life and evolves over decades before the onset of clinical CV events [4]. In recent years, the scope of BHS has expanded beyond the heart to investigate the broader impact of subclinical atherosclerosis on other organ systems, particularly the brain, reflecting the growing recognition that CV health is closely linked to brain health and that early prevention strategies may benefit both [5, 6]. 

Among its most important contributions, the BHS introduced carotid intima–media thickness (cIMT) as a noninvasive, reproducible marker of subclinical atherosclerosis, an approach that was both innovative and rarely applied in pediatric populations at the time. cIMT reflects structural remodeling of the arterial wall and integrates cumulative exposure to CV risk factors over time. Unlike clinical endpoints, which may take decades to manifest, cIMT enables early detection of vascular injury and provides insight into mechanisms linking childhood exposures to adult disease. Over five decades, BHS investigations have examined how obesity, dyslipidemia, blood pressure (BP), and *cardiometabolic clustering* influence arterial structure from childhood into adulthood. In this review, cardiometabolic clustering refers to the co-occurrence of multiple adverse cardiometabolic risk factors within an individual, whose accumulating burden confers progressively greater vascular risk than any single factor alone. More recently, life-course analytic approaches have further clarified the importance of cumulative exposure, developmental timing, and risk factor persistence.

This review synthesizes findings from BHS carotid ultrasound studies, emphasizing methodological evolution, risk factor–specific associations, and life-course patterns of vascular remodeling.

## Evolution of Vascular Assessment in the Bogalusa Heart Study

The earliest vascular imaging studies in the BHS among school-aged children focused on arterial function rather than structure. Using ultrasound techniques available in the 1980s, investigators assessed carotid artery elasticity rather than wall thickness by measuring changes in arterial diameter during the cardiac cycle. Results showed that reduced arterial distensibility was associated with CV risk factors in children and influenced by familial risk profiles [7]. 

Despite their significance and innovation at the time, elasticity-based approaches had limitations. The technique measured vascular function but not arterial structure, meaning it could detect changes in arterial stiffness but could not directly visualize structural alterations in the arterial wall, such as intimal thickening, medial hypertrophy, or early plaque formation. Additionally, early ultrasound technology lacked the resolution made it difficult to quantify the extent of subclinical atherosclerosis or differences across vascular segments.

The introduction of high-resolution B-mode ultrasonography enabled direct visualization and measurement of cIMT, allowing investigators to assess structural arterial remodeling and link early-life risk factors to measurable anatomical changes. Modern carotid ultrasound also allows for obtaining measurements across multiple vascular segments, including the common carotid artery (CCA), carotid bulb, and internal carotid artery (ICA). This allowed investigators to conduct a more detailed assessment of regional patterns of vascular remodeling and subclinical disease, evaluate cumulative CV risk exposure, and track the progression of arterial thickening over time at different locations [8–10]. For example, the carotid bulb often exhibited higher IMT values and greater variability than the CCA, likely reflecting increased hemodynamic stress and susceptibility to atherosclerotic changes [8]. 

## Carotid Intima-Media Thickness Measurement and Definitions in the BHS

Across BHS investigations, cIMT has been quantified using several complementary approaches. Most BHS studies used high-resolution ultrasound with participants in the supine position and applied standardized imaging protocols to measure IMT at segment-specific sites, including the CCA, carotid bulb, and ICA, reflecting the understanding that different arterial segments represent distinct stages of vascular remodeling and atherosclerotic development [8–13]. cIMT measurements are primarily derived from the far wall of each carotid segment and averaged across repeated measurements to improve precision and capture localized arterial wall thickening. Among these segments, the carotid bulb consistently shows the highest absolute IMT values and a distribution skewed toward higher values, consistent with its location at the bifurcation, where flow separation and low, oscillatory shear stress promote early arterial remodeling and make it a particularly sensitive site for detecting subclinical disease in young populations [8]. 

Several complementary approaches have been used to define *cIMT outcomes*. A common method involves calculating the mean of the maximum IMT values from the *right* and *left carotid segments* to assess the impact of clustered cardiometabolic risk factors and familial CV risk on arterial structure in young adulthood [12]. Similarly, studies examining the long-term consequences of childhood obesity have used standardized B-mode ultrasound protocols across these same segments to assess the vascular effects of early-life metabolic exposures [13]. Many BHS analyses also use a *composite cIMT* measure, typically defined as the mean of maximal IMT values across bilateral segments [5, 9, 10, 12–14]. This approach provides a global index of subclinical atherosclerotic burden by integrating information across segments and is interpreted as reflecting overall arterial wall thickening rather than focal plaque formation. In addition, several studies define high cIMT using percentile-based thresholds, most commonly the ≥ 90th percentile, to identify individuals with advanced subclinical atherosclerosis relative to age-, sex-, and race-specific distributions [15, 16]. 

A smaller subset of investigations has focused on *longitudinal change in cIMT* and has also used it as an *exposure* to examine how subclinical arterial changes relate to subsequent health outcomes. Studies have examined progression in segment-specific cIMT over time to identify determinants of arterial remodeling during early adulthood [4, 11, 17, 18]. Higher cIMT and greater progression over time have been linked to later-life CV risk and, more recently, brain health outcomes, including cognitive function [5, 19, 20]. This dual role, as an exposure and outcome measure, strengthens the utility of cIMT as a life-course marker that bridges early cardiometabolic risk with clinically relevant outcomes later in life.

Although these definitions differ slightly in their construction, they capture complementary aspects of subclinical vascular disease, including both the burden and progression of arterial wall thickening.

## Histologic Evidence and the Life-Course Model

Autopsy studies from the BHS provide some of the first and strongest evidence that atherosclerosis starts in childhood and advances throughout life, as well as a crucial biological basis for interpreting cIMT results. These investigations, conducted in children, adolescents, and young adults, demonstrated that atherosclerotic lesions, like fatty streaks and fibrous plaques, are present in nearly all individuals. For instance, coronary involvement increases from ~ 50% in childhood to ~ 85% by young adulthood, accompanied by a marked rise in fibrous plaques with age [4]. In addition, results showed that traditional CV risk factors measured prior to death (elevated body mass index (BMI), BP, and adverse lipid profiles) were strongly associated with the presence and severity of aorta and coronary artery lesions and increased with risk factor clustering, with individuals with ≥ 3 risk factors exhibiting up to 8–12-fold greater coronary lesion extent compared to those with none [4, 21]. Identifying features of progression-prone lesions, including lipid accumulation and structural arterial changes in young individuals, coupled with longitudinal analyses, support the idea that early-life exposures shape vascular health trajectories over decades [21]. 

Overall, these innovative autopsy and longitudinal studies were crucial in establishing that atherosclerosis is a lifelong disease that gradually develops into serious CV disease.

## Life-Course Cardiovascular Risk and Carotid Intima-Media Thickness

### Obesity

Obesity has been among the most consistent and influential determinants of cIMT in the BHS. In this review, adiposity refers to the degree of body fat accumulation, operationalized using body mass index (BMI) as a measure of overall adiposity and waist circumference as a measure of abdominal (visceral) fat distribution. Evidence from cross-sectional and longitudinal designs shows that these measures are consistently and gradually associated with carotid arterial wall thickening. As discussed later, the vascular impact of adiposity is further shaped by metabolic context, with the co-occurrence of other cardiometabolic abnormalities amplifying its atherogenic consequences.

 In a cross-sectional analysis of 518 asymptomatic young adults (mean age ~ 32 years), both BMI and waist circumference were positively and significantly associated with cIMT, with the strongest correlations observed at the common carotid and carotid bulb segments. [8] In multivariable models, BMI remained an independent predictor of internal cIMT, accounting for approximately 5% of its variance, indicating that excess adiposity contributes to arterial wall thickening at this segment beyond the effects of BP and lipids. [8] Across the sample, the carotid bulb showed the highest absolute IMT values and a frequency distribution skewed toward higher values, meaning that a greater proportion of participants had elevated IMT at the bulb than at the other segments. This pattern reflects the bulb’s location at the carotid bifurcation, a region of flow separation and low, oscillatory shear stress that promotes early atherogenesis, making it a particularly sensitive site for detecting subclinical arterial changes in young adults. [8] Together, these findings establish excess adiposity as one of the strongest correlates of early arterial remodeling in young adulthood.

Beyond cross-sectional associations, the BHS evaluated the temporal and cumulative impact of obesity on vascular structure through life-course analyses incorporating repeated BMI measurements from childhood, adolescence, and young adulthood. A one standard deviation higher cumulative BMI was associated with significantly greater adult cIMT in both White (β = 25.5 μm; 95% CI: 14.0–37.1 μm) and Black participants (β = 37.5 μm; 95% CI: 18.3–56.8 μm) after adjustment for physical activity, smoking, alcohol use, and socioeconomic status [22]. The use of a Bayesian relevant life-course model allowed quantification of the relative contribution of BMI at different developmental stages, revealing that young adult BMI contributed most strongly to adult cIMT among White participants, whereas childhood BMI was the dominant contributor among Black participants [22]. These race-stratified findings support both an accumulation model of sustained adiposity-related arterial remodeling and subgroup-specific sensitive periods, suggesting that early-life adiposity may initiate vascular changes with particularly lasting effects in certain populations.

#### Metabolic Context and Obesity

The vascular impact of obesity in the BHS is further modified by metabolic phenotype. Children classified as metabolically healthy obese (MHO) did not exhibit increased adult cIMT compared with non-overweight peers, whereas metabolically abnormal obese children demonstrated significantly higher adult cIMT (*P* = 0.003) [13]. These findings indicate that excess adiposity alone does not uniformly result in vascular injury; rather, concurrent metabolic dysregulation with dyslipidemia, elevated BP, and impaired glucose homeostasis amplifies its atherogenic consequences.

This pattern raises the question of whether adiposity is itself atherogenic or mainly a marker of the metabolic disturbances that accompany it, and BHS evidence suggests it is both. Excess adiposity appears to act largely as an upstream driver whose vascular effects are realized through the abnormalities it commonly produces, including dyslipidemia, elevated BP, insulin resistance, and reduced adiponectin; accordingly, the childhood BMI to adult cIMT association was significant only among adults with below-median adiponectin [23], and removing BMI from composite metabolic definitions attenuates clustering to cIMT associations, indicating that adiposity carries much of that signal [24]. At the same time, adiposity is not merely a passive marker, since cumulative and childhood BMI independently predict adult cIMT after adjustment for concurrent risk factors [4, 22]. This dual role explains why the BMI to cIMT association attenuates, but does not disappear, once metabolic mediators are taken into account. Notably, the metabolically healthy obese phenotype may not confer lasting protection, as it can shift to a metabolically unhealthy state over time; in the BHS it tended to persist into adulthood, though its long-term stability remains uncertain [13]. 

Altogether, BHS evidence positions obesity as a consistent, independent, and modifiable determinant of subclinical atherosclerosis across the life course. Cross-sectional data establish strong associations between adiposity and carotid wall thickness in young adulthood [8], life-course modeling demonstrates that cumulative BMI exposure contributes independently to adult cIMT with race-specific patterns [22], and metabolic phenotype analyses confirm that cardiometabolic abnormalities amplify the vascular consequences of excess adiposity [13]. Mechanistically, obesity promotes arterial remodeling through insulin resistance, chronic inflammation, endothelial dysfunction, and increased hemodynamic load [25]. These findings underscore the importance of minimizing excess adiposity and its metabolic sequelae beginning in childhood to limit the progression of subclinical atherosclerosis.

### Lipids

Dyslipidemia, particularly elevated low-density lipoprotein cholesterol (LDL-C), is consistently associated with increased cIMT in the BHS. LDL-C has been identified as a strong independent predictor of carotid wall thickness across arterial segments, while HDL-C demonstrates inverse associations [3, 8]. 

#### Cross-Sectional Associations Between Lipids and cIMT

Cross-sectional BHS analyses establish LDL-C and HDL-C as the principal *lipid* determinants of early carotid wall thickening in young adulthood, acting through opposing mechanisms: LDL-C promotes cholesterol deposition in the arterial intima, while HDL-C participates in reverse cholesterol transport and is inversely associated with wall thickness. In foundational segment-specific analyses, LDL-C was independently associated with both CCA and carotid bulb IMT, while HDL-C demonstrated an inverse independent association; triglycerides correlated in bivariate analyses but did not emerge as independent predictors, indicating that the balance between atherogenic and protective lipid fractions is more informative than triglycerides alone for subclinical carotid thickening at this age [8]. In this young, largely lesion-free population, the thickening reflects early atherogenic changes in the arterial wall rather than the medial hypertrophy that predominates with elevated BP; consistent with this intimal, lipid-driven contribution, the total cholesterol/HDL-C ratio consistently predicted cIMT across carotid segments in multiple BHS analyses, positioning dyslipidemia as a persistent, independent contributor even as SBP remained the strongest overall determinant [9, 10]. 

#### Life-Course and Cumulative Lipid Exposure

Longitudinal analyses demonstrate that LDL-C is the dominant lipid fraction predicting adult cIMT, with cumulative exposure showing the strongest associations. In studies linking repeated measures from childhood into young adulthood, childhood LDL-C predicted higher odds of elevated adult cIMT, adult LDL-C was independently predictive, and cumulative LDL-C burden showed the strongest overall association. HDL-C demonstrated inverse associations in adulthood and cumulative models, while triglycerides did not persist as independent predictors [3]. Extending these findings into midlife, life-course lipid trajectory analyses showed that lipid levels at each age from childhood through adulthood were associated with later cIMT, with non–HDL-C and LDL-C showing the strongest and most consistent associations and effect sizes generally strengthening with advancing age, supporting the importance of both early identification and sustained maintenance of optimal lipid profiles across decades [26]. 

#### Developmental Timing and Adolescence as a Critical Window

A key pattern across BHS and BHS-linked International Childhood Cardiovascular Cohort Consortium (i3C) analyses is that the predictive relevance of lipids for adult cIMT strengthens beginning in later childhood and adolescence. In pooled i3C analyses, associations between childhood risk factors and adult cIMT were notably stronger when measured at age ≥ 9 years, supporting adolescence as a more informative window for predicting later subclinical atherosclerosis [27]. Complementing this, adolescents with dyslipidemia were at significantly higher risk of high adult cIMT (≥ 90th percentile), with risk further amplified in the presence of overweight or obesity [28]. The timing signals identified are consistent with i3C metabolic syndrome analyses showing that lipid components predicted adult metabolic syndrome and high cIMT, particularly during later adolescence, though BMI tended to be a more consistent predictor of elevated adult cIMT than lipids alone, underscoring that dyslipidemia often operates within broader cardiometabolic phenotypes [24]. While universal pediatric lipid screening is primarily aimed at detecting familial hypercholesterolemia [29], these findings underscore that adolescents with concurrent adiposity and dyslipidemia represent a distinct high-risk group in whom lipid assessment carries added prognostic value for subclinical atherosclerosis, and in whom lifestyle-based intervention may be particularly beneficial.

#### Tracking, Persistence, and Mediation by Adult Lipid Levels

BHS analyses suggest that lipid effects on cIMT reflect both early-life exposure and the persistence of dyslipidemia into adulthood, rather than independent childhood effects. It is important to remember that cIMT does not measure atherosclerotic plaque directly; rather, it captures diffuse arterial wall thickening that precedes and accompanies plaque formation, reflecting both early lipid-driven intimal remodeling and the chronic vascular inflammation and endothelial dysfunction that dyslipidemia promotes. LDL-C contributes to both processes, driving cholesterol deposition into the arterial intima and the inflammatory response that thickens the wall well before focal lesions are detectable. In analyses using cumulative exposure metrics, childhood lipid levels were associated with adult cIMT; but these associations were substantially attenuated after accounting for adult lipid levels, indicating that the tracking and persistence of dyslipidemia into adulthood likely mediate much of the observed relationship [14]. Nonetheless, lipid balance metrics such as the total cholesterol/HDL-C ratio, a more consistent correlate of cIMT than any single lipid fraction, remained independently associated with composite and segment-specific cIMT after multivariable adjustment in certain model contexts, confirming that dyslipidemia can contribute directly to carotid wall thickening beyond covarying risk factors [10], an observation subsequently reinforced in independent populations [30, 31]. 

#### Lipids and cIMT Progression

When the focus shifts from cumulative cIMT burden to short-term progression, lipid findings are more mixed. In segment-specific progression analyses of young adults over a mean 2.4-year follow-up, age and mean arterial pressure were the most consistent independent predictors across all three carotid segments, joined by smoking, insulin resistance (HOMA-IR), or waist/height ratio at the bulb and internal segments [18]. Among lipid measures, higher HDL-C was inversely associated with common carotid IMT progression, but was not an independent predictor at the bulb or internal segments, and LDL-C and triglycerides did not independently predict progression at any segment after multivariable adjustment [18]. These findings do not establish HDL-C as a directly protective agent; the inverse association is more plausibly interpreted as reflecting HDL-C’s role as a marker of overall atherogenic lipoprotein burden, consistent with contemporary evidence that HDL-C levels themselves do not causally modify atherosclerosis [32, 33]. In a separate young-adult progression analysis, lipid fractions and cholesterol ratio were similarly not independent predictors of cIMT progression, indicating that lipids are more strongly linked to cumulative cIMT burden than to short-interval arterial change, particularly when BP and smoking are accounted for [11]. 

LDL-C and the total cholesterol/HDL-C ratio are consistently identified as the principal lipid determinants of carotid wall thickening, while triglycerides show limited independent predictive value. Cumulative LDL-C exposure carries the strongest association with adult cIMT, with associations strengthening from childhood through midlife and amplified in the presence of obesity. Much of the childhood lipid effect appears to be mediated by the persistence of dyslipidemia into adulthood, positioning sustained lipid burden, rather than any single exposure window, as the key driver of subclinical atherosclerosis in this cohort.

### Blood Pressure

#### Cross-Sectional Associations Between Blood Pressure and cIMT

Across BHS investigations, systolic blood pressure (SBP) emerges as the most consistent hemodynamic determinant of cIMT in cross-sectional analyses of young adults, frequently exceeding lipid variables in explanatory strength when modeled together. In foundational segment-specific analyses, SBP was the strongest independent predictor of CCA IMT and a major contributor to carotid bulb IMT, accounting for the largest share of variance in composite cIMT relative to all other risk factors [9]. Diastolic blood pressure (DBP) showed weaker and less consistent relationships, suggesting that pulsatile hemodynamic load is more relevant to early arterial wall thickening than steady-state pressure alone [8–10]. However, while these cross-sectional analyses indicate that SBP is the dominant single predictor of cIMT level, obesity-related measures also emerged as strong and independent contributors, with the two risk factors often operating in tandem.

#### Life-Course and Cumulative Blood Pressure Exposure

Life-course analyses provide important nuance on when and how BP contributes to cIMT, and how it compares with obesity across developmental windows. In the landmark JAMA BHS analysis directly comparing childhood, adulthood, and cumulative risk factor burdens, adulthood SBP independently predicted high cIMT, whereas childhood SBP did not remain an independent predictor after multivariable adjustment, in contrast to childhood BMI and LDL-C, which did remain significant childhood predictors [3]. This pattern suggests that for BP, contemporaneous adult hemodynamic load may exert a stronger influence on carotid thickening than early-life exposure alone, while for obesity, childhood exposure carries independent predictive value beyond adult levels. Consistent with this interpretation, cumulative childhood SBP and DBP correlated with adult cIMT in unadjusted analyses but were attenuated after adjustment for adult BP levels, whereas cumulative adult BP showed a stronger association. This reinforces that persistently elevated BP into adulthood mediates much of the BP–cIMT relationship [34]. 

An additional interpretive consideration on bidirectional relationships, most relevant to later life stages, is that cumulative lifetime exposure to atherogenic lipoproteins, particularly LDL-C, may contribute to the development of subclinical atherosclerosis, which is one of several contributors to arterial stiffening, that in turn promotes the elevated systolic BP observed in adulthood [35, 36]. In BHS, a cross-lagged path analysis of middle-aged participants found that baseline BP was a significantly stronger predictor of follow-up aortic-femoral pulse wave velocity than baseline pulse wave velocity was of follow-up BP, and the authors explicitly noted that, unlike in older adults, the large-arterial wall in young and middle-aged BHS participants is not yet stiff enough to alter BP levels [37]. Consistent with this, a 25-year analysis of the Coronary Artery Risk Development in Young Adults (CARDIA) cohort demonstrated that greater cumulative BP exposure during young adulthood was independently associated with both higher cIMT and coronary artery calcium in middle age, providing better prognostic value than a single baseline BP measurement [38]. These findings position BP as an upstream driver of subclinical atherosclerosis and arterial remodeling from young through middle adulthood; the balance of causal direction may shift at later life stages, when accumulated vascular changes can in turn contribute to BP elevation; however, the pathway end-to-end within a single cohort is currently lacking, reflecting the scarcity of studies that follow the natural progression from childhood exposure through subclinical atherosclerosis to incident hypertension, and remains an important area for investigation.

#### Blood Pressure Within Cardiometabolic Clustering

When examined as part of broader cardiometabolic clustering, in young adults, BP consistently emerges as the most influential single component for cIMT, while obesity appears to serve as the central organizing factor around which other risks tend to cluster. Metabolic syndrome was associated with higher CCA and ICA IMT, and in component-level modeling, the elevated BP component showed the strongest independent association with composite cIMT, identifying hemodynamic load as the dominant metabolic syndrome contributor to subclinical carotid atherosclerosis in this age range [39]. Across the life course, however, i3C consortium analyses showed that elevated childhood BP predicted adult metabolic syndrome and was associated with later high cIMT at certain ages, but its predictive strength for adult cIMT was less consistently robust than BMI, further reinforcing that while BP is a powerful hemodynamic driver in young adulthood, obesity may be a more consistent upstream determinant of subclinical atherosclerosis when the entire developmental window is considered [24]. 

#### Blood Pressure and cIMT Progression

In progression analyses over short follow-up intervals, SBP independently predicted ICA and composite cIMT progression in men, with weaker and borderline evidence in women, meaning that hemodynamic load may contribute more strongly to short-term arterial wall thickening in young men within this cohort [11]. Taken together, BHS findings position SBP as the primary hemodynamic determinant of cIMT in cross-sectional analyses, while life-course evidence suggests that obesity and BP are comparably important drivers of subclinical atherosclerosis over time. SBP dominates in adult models, and BMI shows more consistent independent effects beginning in childhood. Neither risk factor fully eclipses the other; rather, their combined and sustained burden across the life-course appears to be the most relevant determinant of early arterial remodeling [3, 9, 18]. 

## Metabolic Syndrome and Risk Factor Clustering

### Metabolic Syndrome and Cardiometabolic Clustering in Young Adults

In the primary BHS MetS analysis of 507 non-diabetic young adults (mean age ~ 32 years), MetS prevalence was approximately 13% under both NCEP (National Cholesterol Education Program) and WHO criteria. Participants meeting MetS criteria had significantly greater CCA and ICA IMT, as well as higher composite cIMT, compared with individuals without MetS, with associations observed under both diagnostic frameworks, though slightly attenuated under the WHO definition [39]. Importantly, composite cIMT increased progressively with the number of MetS components present under both definitions, supporting a graded, dose–response relationship between cardiometabolic burden and early arterial wall thickening in otherwise asymptomatic young adults.

Beyond formal MetS definitions, BHS analyses using a continuous clustering approach similarly demonstrate that the accumulation of adverse cardiometabolic traits is associated with progressively greater carotid IMT. In analyses incorporating five adverse factors, elevated waist circumference, SBP, LDL-C, insulin, and smoking, mean cIMT increased stepwise with the number of risk factors present, particularly for CCA and carotid bulb segments, with those carrying three or more adverse factors exhibiting the highest IMT values [8]. 

### Life-Course and Childhood Clustering

Life-course analyses provide evidence that cardiometabolic clustering beginning in childhood predicts later vascular changes, with an important caveat regarding the role of adiposity. Results from i3C consortium analyses showed that childhood MetS, defined using age-specific percentile thresholds, was associated with an approximately two- to four-fold increased risk of high adult cIMT when assessed beginning in adolescence (ages ~ 11–18 years) [24]. Critically, when BMI (the adiposity measure used in these analyses) was removed from the MetS definition, associations with adult high cIMT were attenuated, indicating that BMI substantially drives the vascular risk associated with childhood cardiometabolic clustering [24]. Consistent with these findings, analyses of composite childhood risk scores (total cholesterol, triglycerides, BMI, and SBP) demonstrated that higher cumulative cardiometabolic burden predicted high adult cIMT beginning around age nine, with associations strengthening across adolescence [27]. A notable finding, first demonstrated in the combined BHS and Young Finns analysis [40] and later confirmed in the broader i3C consortium [24], is that pediatric MetS predicted adult high cIMT, adult MetS, and type 2 diabetes but performed no better than BMI alone as a screening tool. This means that adiposity explains much of the predictive value of childhood MetS for adult vascular risk, with the other clustering components adding only modest incremental information; accordingly, the simplicity of elevated BMI may be an equally informative and more practical indicator of vascular risk in youth.

### Familial Risk as a Modifier

BHS analyses further indicate that genetic susceptibility amplifies the vascular consequences of cardiometabolic clustering. In young adults, a parental history of coronary artery disease (CAD) strengthened the association between MetS and cIMT, with more pronounced effects observed in specific vascular segments, particularly the ICA [12]. A plausible hypothesis for this pattern is that the ICA experiences hemodynamic conditions at the bifurcation, such as flow separation and low oscillatory shear stress, that predispose it to early atherosclerotic formation and make it particularly sensitive to both inherited susceptibility and cumulative cardiometabolic exposure [41]. This anatomical vulnerability is consistent with the ICA being a primary site of clinically significant atherosclerotic disease, and with ICA IMT being more strongly associated with future stroke and myocardial events than CCA IMT in adult cohorts [42]. These findings demonstrate that familial CV risk modifies the impact of cardiometabolic clustering on subclinical atherosclerosis, suggesting that inherited susceptibility may exacerbate clustering-related vascular injury and increase vulnerability to early arterial remodeling, particularly at hemodynamically vulnerable sites.

### Metabolic Health Status and the Obesity–cIMT Relationship

Phenotype-based analyses classify individuals by combinations of weight status and metabolic profile, that is, the presence or absence of cardiometabolic abnormalities (e.g., elevated BP, dyslipidemia, impaired glucose metabolism) rather than weight alone. Following this approach, BHS analyses demonstrate that metabolic health status critically modifies the vascular impact of childhood obesity, reinforcing themes discussed in the Obesity section. Children classified as metabolically abnormal obese (elevated VMI with cardiometabolic abnormalities) exhibited the highest adult cIMT values, whereas metabolically healthy overweight children (elevated BMI with no cardiometabolic abnormalities) did not differ significantly from metabolically healthy normal-weight peers in adult cIMT [13]. Clustering of cardiometabolic abnormalities within obesity, rather than excess adiposity alone, is the primary driver of early subclinical atherosclerosis, consistent with the layered risk model described earlier.

Taken together, BHS findings consistently demonstrate that MetS and related cardiometabolic clustering constructs represent integrative markers of cumulative CV risk, associated with greater cIMT in young adulthood and identifiable beginning in childhood and adolescence. The clustering impact is amplified by familial CV risk and is heavily influenced by whether obesity occurs alongside other metabolic abnormalities.

## Limitations

Several limitations of the BHS cIMT literature warrant consideration. First, cIMT measurements were not obtained during adolescence in the early phases of the study, precluding direct evaluation of arterial changes during this potentially critical developmental window. Second, longitudinal cIMT progression analyses are limited to relatively short follow-up intervals in young adulthood, restricting conclusions about the long-term trajectory of arterial remodeling. Third, because of the participants’ age, there is a low rate of clinical CV events within the BHS cohort, which limits the evaluation of cIMT as a predictor of hard outcomes such as myocardial infarction or stroke. Fourth, cIMT definitions and measurement protocols varied across studies, which introduces some heterogeneity in comparisons across analyses. Fifth, residual confounding by unmeasured factors, including diet quality, physical activity, and psychosocial stress, cannot be excluded in observational analyses. Finally, although BHS has enrolled both Black and White participants, the cohort is drawn from a single rural community in Louisiana, which may limit generalizability to other racial, ethnic, or geographic populations.

## Conclusions

The BHS provides over five decades of compelling evidence that atherosclerosis is a lifelong process rooted in childhood risk factor exposure and measurable through cIMT well before clinical disease appears. Obesity, dyslipidemia, and SBP, primarily, each contribute independently to carotid wall thickening, and the clustering of these factors within individuals amplifies their impact, such that their combined and sustained burden across the life course represents the strongest determinant of subclinical arterial remodeling. Life-course analyses have clarified that when risk-factor exposure in integrated across decades, cumulative BMI and LDL-C exposure carry the greatest predictive value for adult cIMT, with race-specific sensitive periods underscoring the importance of timing and context. BP contributed differently across life stages: SBPis the strongest hemodynamic predictor of cIMT in cross-sectional analyses of young adult, while cumulative BMI is the most consistent longitudinal predictor when childhood exposure is followed into adulthood, so the relative importance of these risk factors depends on the analytic window rather than one uniformly outweighing the other. Adolescence emerges as a critical window for risk stratification, particularly for dyslipidemia in the context of obesity, though the simplicity of BMI as a screening tool rivals more complex clustering constructs. The extension of BHS findings to brain health outcomes further elevates cIMT as a life-course marker linking early cardiometabolic risk to cognitive function in midlife. Collectively, these findings provide compelling support for early, sustained, and multifactorial prevention strategies targeting modifiable CV risk factors beginning in childhood, with particular emphasis on adiposity, lipid control, and BP management across the lifespan.

## Key References

- Evans JT, Buscot MJ, Fraser BJ, Juonala M, Guo Y, Fernandez C, et al. Life-period associations of body mass index with adult carotid intima-media thickness: The Bogalusa Heart Study and the Cardiovascular Risk in Young Finns Study. Prev Med. 2024;189:108128. https://doi.org/10.1016/j.ypmed.2024.108128.
  - ○ This pooled BHS and Young Finns Study analysis using a Bayesian life-course model quantified the relative contributions of BMI at childhood, adolescence, and young adulthood to adult cIMT, revealing race-specific sensitive periods and supporting a cumulative exposure model of obesity-related arterial remodeling.
- Yan Y, Li S, Liu Y, Guo Y, Fernandez C, Bazzano L, et al. Associations Between Life-Course Lipid Trajectories and Subclinical Atherosclerosis in Midlife. JAMA Netw Open. 2022;5(10):e2234862. https://doi.org/10.1001/jamanetworkopen.2022.34862.
  - ○ This BHS-based life-course lipid trajectory analysis extending into midlife demonstrated that non–HDL-C and LDL-C showed the strongest and most consistent associations with later cIMT across all ages from childhood through adulthood, with effect sizes strengthening with advancing age. These findings reinforce the clinical importance of sustained lipid control beginning in childhood.
- De Anda-Duran I, Alonso CF, Libon DJ, Carmichael OT, Kolachalama VB, Suglia SF, et al. Carotid Intima-media Thickness and Midlife Cognitive Function: Impact of Race and Social Disparities in the Bogalusa Heart Study. Neurology. 2022. https://doi.org/10.1212/WNL.0000000000200155.
  - ○ This BHS cross-sectional study demonstrated that cIMT above the 50th percentile was independently associated with worse midlife cognitive function, with stronger effects in Black participants and those with lower education. It extends the clinical significance of cIMT beyond cardiovascular endpoints to brain health outcomes.
- Koskinen J, Magnussen CG, Sinaiko A, Woo J, Urbina E, Jacobs DR, et al. Childhood Age and Associations Between Childhood Metabolic Syndrome and Adult Risk for Metabolic Syndrome, Type 2 Diabetes Mellitus and Carotid Intima Media Thickness: The International Childhood Cardiovascular Cohort Consortium. J Am Heart Assoc. 2017;6(8):e005632. https://doi.org/10.1161/JAHA.117.005632.
  - ○ This i3C consortium analysis including BHS showed that childhood metabolic syndrome predicts adult high cIMT beginning in adolescence (ages 11–18), but performs no better than BMI alone. This practically important finding supports the use of simple BMI-based screening as an efficient tool for identifying youth at vascular risk.

## Data Availability

No datasets were generated or analysed during the current study.
